# High-pressure single-crystal diffraction at the Australian Synchrotron

**DOI:** 10.1107/S160057752300406X

**Published:** 2023-06-15

**Authors:** Stephanie A. Boer, Jason R. Price, Alan Riboldi-Tunnicliffe, Rachel Williamson, Robert Rostan, Aston Summers, Gemma F. Turner, Isabelle Jones, Charles S. Bond, Alice Vrielink, Andrew C. Marshall, John Hitchings, Stephen A. Moggach

**Affiliations:** a Australian Synchrotron, 800 Blackburn Rd, Clayton, Melbourne, Victoria 3168, Australia; b School of Molecular Sciences, The University of Western Australia, 35 Stirling Highway, Crawley, Perth, Western Australia 6009, Australia; c Peakwell Systems, Australia; Advanced Photon Source, USA

**Keywords:** pressure, synchrotron, single crystal

## Abstract

A new high-pressure single-crystal diffraction setup has been designed and implemented at the Australian Synchrotron for collecting molecular and protein crystal structures. The setup incorporates a modified micro-Merrill–Bassett cell and holder designed specifically to fit onto the horizontal air-bearing goniometer, allowing high-pressure diffraction measurements to be collected with little to no modification of the beamline setup compared with ambient data collections. Compression data for the amino acid, l-threonine, and the protein, hen egg-white lysozyme, were collected, showcasing the capabilities of the setup.

## Abstract 2

A new high-pressure single-crystal diffraction setup has been designed and implemented at the Australian Synchrotron for collecting molecular and protein crystal structures.

## 1. Introduction

**Figure 1:**
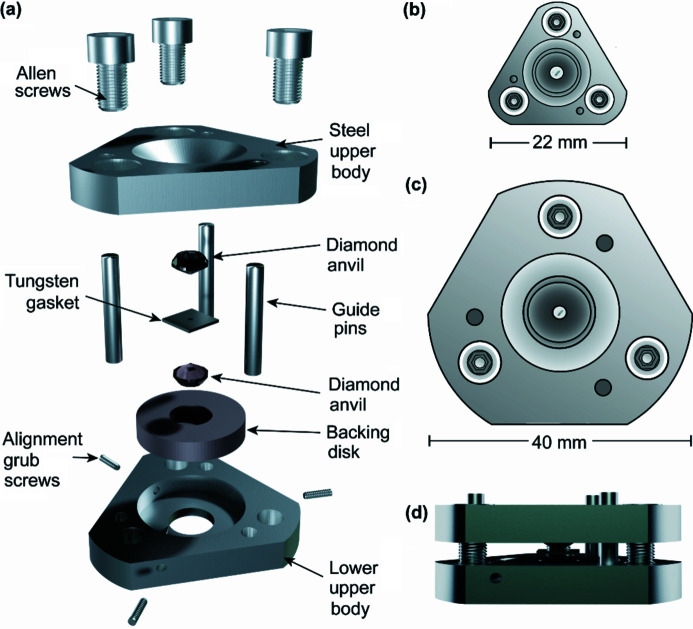
Rendered images of the micro-Merrill–Bassett (MMB) cell designed for MX1 shown as (*a*) a blown-up diagram with steel body, diamond anvils, backing disk, gasket and grub screws for horizontal alignment, (*b*) a top view with the MMB cell diameter, (*c*) a top view of a miniature-Merrill–Bassett cell with cell diameter to highlight the size comparison, and (*d*) a rendered view of the MMB, showing the closed cell.

High-pressure single-crystal diffraction techniques have been well developed for the investigation of materials over a wide range of scientific disciplines. Recent examples of materials where structure elucidation has been performed at pressure include spin-crossover complexes (Turner *et al.*, 2020 ▸), molecular magnets (Etcheverry-Berrios *et al.*, 2020 ▸), pharmaceuticals (Oswald *et al.*, 2010 ▸), molecular and framework porous materials (McKellar & Moggach, 2015 ▸), gases (Lundegaard *et al.*, 2009 ▸), pure metals (McMahon & Nelmes, 2006 ▸), alloys (Perez-Albuerne *et al.*, 1966 ▸), proteins (Librizzi *et al.*, 2018 ▸) and molecules associated with planetary science (Cable *et al.*, 2021 ▸). A range of high-pressure single-crystal diffraction apparatuses have been developed over the years for investigating these materials, including high-pressure capillary pressure cells for both laboratory diffractometers (Yufit & Howard, 2005 ▸) and for use at central facilities (McMonagle *et al.*, 2020 ▸) for exploring the effect of lower pressures (<2000 atm) on soft materials (McMonagle *et al.*, 2022 ▸). By far the most common high-pressure single-crystal diffraction apparatus utilized to apply higher pressures (>2000 atm, 0.2 GPa) is the diamond anvil cell (or DAC) (Merrill & Bassett, 1974 ▸; Moggach *et al.*, 2008 ▸). DACs are generally composed of a body made from stainless steel (or high-tensile Vascomax steel), backing disks, diamond anvils and a gasket material (Fig. 1 ▸). The diamonds have a small flat surface ground onto them (referred to as the culet), which varies in size but is generally of the order of a few hundred micrometres. The sample chamber is formed as a cylindrical hole (with a diameter smaller than the diamond culet) drilled through a gasket, usually made from tungsten, Inconel or steel, which is placed between the opposed diamond anvils (Dustan, 1989 ▸). A single crystal or polycrystalline powder can be housed in the sample chamber, along with a pressure-transmitting medium, which is usually a liquid or soft solid (Tateiwa & Haga, 2010 ▸). Ruby is also placed inside the cell to act as an internal pressure calibrant, with the ruby fluorescence method utilized to measure the pressure (Barnett *et al.*, 1973 ▸). Other diffraction calibrants have also been used, including quartz and lead (Angel *et al.*, 1997 ▸; Strässle *et al.*, 2014 ▸).

Implementation of data collection and reduction procedures have been well established on laboratory instruments, with most diffractometer manufacturers highlighting this capability in their instruments. Most are optimized for data collection of Merrill–Bassett style DACs as they are small enough to fit on laboratory diffractometers, albeit with some slight modifications, including a shorter collimator and extended beamstop. High-pressure single-crystal diffraction has also been well developed at numerous central facilities across the world, including both synchrotron and neutron scattering facilities. Examples include beamline I19 at Diamond Light Source (UK) (Nowell *et al.*, 2012 ▸), GSECARS 13-BM-C beamline at the Advanced Photon Source (USA) (Zhang *et al.*, 2017 ▸, 2022 ▸; Shen *et al.*, 2005 ▸) and the BL10XU beamline at SPring-8 (Japan) (Hirao *et al.*, 2020 ▸; Utsumi *et al.*, 2002 ▸; Watanuki *et al.*, 2001 ▸). These beamlines are dedicated to extreme conditions and feature large goniometers and spacious experimental hutches capable of accommodating Merrill–Bassett DACs or other environment cells.

Here we present a new high-pressure single-crystal diffraction setup at the MX1 beamline at the Australian Synchrotron that does not require modification to the existing configuration (Cowieson *et al.*, 2015 ▸). The setup uses a micro-Merrill–Bassett (MMB) cell (22 mm maximum diameter) at other synchrotrons; the technique can be adapted to any beamline without the need for a dedicated high-pressure beamline.

The MX1 beamline operates between a user-defined range of 8–18 keV (λ = 0.69–1.77 Å) with a double-crystal Si(111) monochromator (Cowieson *et al.*, 2015 ▸). A Kirkpatrick–Baez bimorph mirror focuses the beam in the vertical and horizontal directions to a size of 120 µm × 120 µm (FWHM) (Cowieson *et al.*, 2015 ▸; Siewert *et al.*, 2012 ▸). The mirror and camera (Navitar zoom and focus camera) are positioned at 45° and 90°, respectively, to the beam such that the sample is viewed on-axis and can be centred by ‘click-to-centre’ motorized stages that adjust the focus and move the sample in lateral and vertical positions. The MX1 beamline is equipped with a mini-κ goniometer head (Brockhauser *et al.*, 2013 ▸), allowing diffraction data to be collection in six ω-scans of 70° in three settings of φ and κ. This gives high-pressure data to a completeness of 45% of the structure of l-threonine modelled in space group *P*2_1_2_1_2_1_, for example. A Dectris EIGER2 9M photon-counting detector permits fast data collection. High-pressure diffraction data on two materials, namely the amino acid, l-threonine, and the protein, tetragonal hen egg-white lysozyme (tHEWL), are used to demonstrate the utility, speed and quality of the high-pressure data that can be collected.

## 2. Diamond anvil cell and mount design

**Figure 2:**
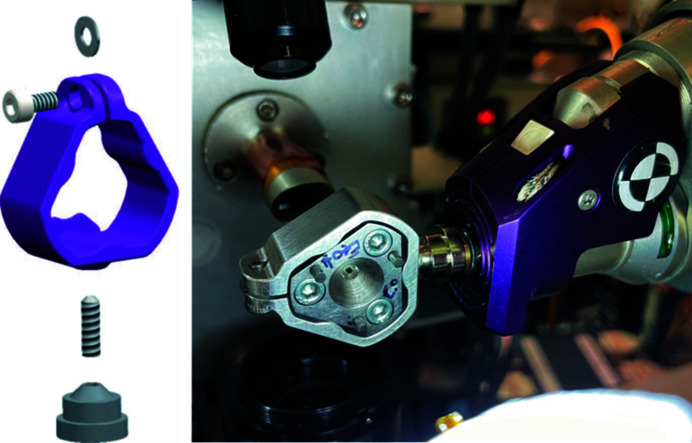
MMB magnetic base-cell holder clamp design and MMB cell mounted onto the MX1 beamline at the Australian Synchrotron.

We have designed and built a small diamond anvil cell (hereafter referred to as the MMB cell), which is similar in design to the original Merrill–Bassett cell except the main mass of the cell body has been reduced, both by decreasing the size of the diameter of the cell body from 40 mm to 22 mm and by removing one of the backing disks while maintaining a full-opening angle of 80° [Figs. 1 ▸(*a*)–1(*c*)]. Although this limits the alignment capability of the diamond anvils, diamond alignment can still easily be obtained by having alignment screws and backing disk on one half of the cell body. The reduction in mass of the cell body was done to mount the cell onto the goniometer on the MX1 beamline at the Australian Synchrotron with no modification of the beamline sample environment required other than the retraction of the open flow cryostat (Fig. 2 ▸). This offers simplicity over other extreme conditions beamlines, where large goniometers and spacious experimental hutches are required to accommodate the heavier pressure cell (Nowell *et al.*, 2012 ▸; McMahon, 2015 ▸; Allan *et al.*, 2017 ▸).

The cell is similar in design to the miniature DAC used by McIntyre and co-workers (Novelli *et al.*, 2022 ▸) for joint X-ray and neutron experiments, although, for ease of production, the cell body and backing disk in the cell constructed and used here is made from cobalt-enriched Grade 420 stainless steel, and not from a beryllium–copper alloy (BERYLCO-25), while the cell body was designed to accommodate Boehler–Almax cut diamond anvils, identical to those incorporated into a standard modified Merrill–Bassett cell, with 4.00 mm, 80° (100)-oriented, 16-sided, ±0.33 carat diamond anvils with 600 µm culets. A magnetic-based clamp was designed to both hold the cell and allow the MMB to be mounted onto the goniometer (Fig. 2 ▸). The magnetic base of the clamp is identical to the single-crystal diffraction magnetic base-mounts available from commercial suppliers, and is therefore easily transferable to other beamlines, or laboratory goniometers, if needed. Because of the reduced mass and limited diamond alignment capabilities of the cell, obtaining pressures above ∼5 GPa is unlikely to be achieved; however, the small cell design and ease of mounting onto the goniometer make it ideal for high-throughput high-pressure diffraction on molecular systems.

## 3. MMB cell loading, alignment, data collection, reduction and refinement

To ascertain the efficacy of data collection, reduction and refinement, two test samples were chosen – the amino acid l-threonine and the protein tHEWL. Both were selected as they are highly crystalline, and previous high-pressure studies have been performed on both systems, ensuring no phase transitions or unusual structural behaviour would affect data quality within the pressure region investigated. Crystals of l-threonine were used as received (Sigma Aldrich), while lyophilized chicken egg-white lysozyme was crystallized by dissolving to 40 mg ml^−1^ in 20 m*M* sodium acetate pH 4.7, and grown by vapour diffusion, as described previously (Yamada *et al.*, 2015 ▸).

Prior to data collection, a single crystal of l-threonine was collected under ambient conditions on a laboratory Synergy-S HyPix diffractometer using Mo *K*_α_ radiation. The same crystal was then loaded into an MMB cell using ethanol as a hydro­static liquid at 0.15 GPa and collected on the same Synergy-S HyPix diffractometer to compare with the synchrotron data. Data were collected based on an optimized strategy calculation from the program *CrysAlisPro* (Rigaku Oxford Diffraction, 2019 ▸) for a DAC with a 40° half-opening angle. A crystal of tHEWL was loaded with the mother liquor used to crystallize the sample as the pressure-transmitting medium in a separate MMB cell at 0.44 GPa. Both samples were loaded with ruby spheres, used as an internal pressure calibrant, with the ruby fluorescence method used to measure the pressure. Laboratory data were not collected on tHEWL to minimize beam damage.

The MMB cells were then mounted onto the horizontal air-bearing goniometer on MX1 at the Australian Synchrotron (λ = 0.7109 Å) using the bespoke magnetic holder (Fig. 2 ▸) (Cowieson *et al.*, 2015 ▸) with the flat face of the DAC facing down and the ω-axis set to 90°. A small spirit level was placed on the flat face of the DAC and the magnetic holder adjusted on the goniometer to ensure that the DAC was level and would therefore be perpendicular to the beam direction when ω was 0°.

The ω-axis was then rotated to 0° so that the sample could be optically aligned through the visualization camera. The sample chamber was found and centred via the ‘click-to-centre’ system enabled by motorized stages, so that it is centred on the crosshairs. The *x*-axis of the goniometer, which at ω = 0° (and 180°) moves in the direction of the beam, was adjusted via a graphical user interface (GUI) to bring the DAC sample chamber and the crystal into focus. The position of the *x*-axis (in µm) was noted down in the GUI. The goniometer was then rotated to ω = 180° and the *x*-axis adjusted again to bring the DAC sample chamber and crystal back into focus. This position of the *x*-axis was also noted down in the GUI. The GUI automatically calculates the centre point between these two positions of the *x*-axis, which represents the centre point between the two diamonds of the DAC, and therefore the crystal, lying on the ω rotation axis of the goniometer. The position of the *x*-axis is changed to this middle position. The focal length of the sample camera is then adjusted, also on the user GUI, to bring the sample into focus. This sample centring protocol serves to centre the crystal on the rotation axis of the goniometer while also allowing sample visualization through the sample camera when the ω-axis is at 0° and 180°. The centring procedure may take several minutes.

This sample alignment procedure is necessary because the refractive index of the diamonds results in the focal length changing. However, because the diamond anvils are identical in size, shape and thickness, the change in focal length is identical on both sides of the cell, meaning the sample chamber can be viewed in focus on both sides of the DAC, allowing optical alignment. The wide beam of MX1 meant that imprecise centring on the crystal did not substantially impact the quality of data collection.

Once aligned, data were collected in up to six 70° wedges via rotation of the ω-axis (Table 1 ▸). Due to the large area of the Dectris EIGER2 9M, all data collection was carried out with 2θ of 0°. Data collection was performed with the detector at its closest possible approach to the sample, which is 105 mm from the crystal.

Data completeness is limited by shading of the pressure cell body. Though this could be compensated for by using a shorter wavelength, which compresses the diffraction pattern into a smaller volume, the current wavelength (λ = 0.7109 Å) on the MX1 beamline is close to the accessible limit. To access more redundant data, adjustment of the κ- and φ-axis of the mini-κ goniometer head was made. The first run is a 70° rotation around ω = 0°, such that it rotates from −35°, where half the frame is shaded by the cell body, to 35°, where the other half of the frame is shaded by the cell body. The second run is a 70° rotation around 180°, so it collects data between ω = 145° and 215°. These first two runs were collected via ω-scans with the κ- and φ-axis set to 0. To reorient the crystal to access more data, the κ-axis is then rotated to 180°, which moves the crystal to a χ angle of 48°. The third run is also between ω = −35° and 35°, and the fourth run a collection between ω = 145° and 215°. The fifth and sixth runs occur when the κ-axis is still at 180° and the φ-axis is also set to 180°, which moves the crystal to a χ angle of −48°. These final two runs are also a collection between ω = −35° and 35°, and the fourth run a collection between ω = 145° and 215°. Unfortunately, the movement of the κ- or φ-axes of the mini-κ goniometer results in the loss of centre of the sample – it must be realigned to return the crystal to the centre of rotation of the ω-axis. During the subsequent alignments, it was found to not be necessary to change the value of the camera focus. The *x*-axis is adjusted to bring the crystal into focus at ω = 0° and then ω = 180°, and the *x*-axis is moved to the centre point of these two values, which represents the crystal being at the centre of rotation, and in focus.

The frame step size was 0.1°, collecting 700 frames in the 70° wedge of each of the six runs. In the case of l-threonine, each run had a total collection time of 14 s, so the frame time was 0.02 s. This short exposure time was chosen because the sample was strongly diffracting, and it was found that this was the optimum exposure time for collecting strong diffraction spots without them resulting in overloads on the detector. The chosen step size provided over 1200 reflections for l-threonine, which was sufficient for a reliable structure solution and refinement, while keeping the collection time short. In addition, fine slicing helps to minimize the background whilst allowing better integration of individual reflections over multiple images.

The .h5 files were converted to .cbf files using *eiger2cbf* (https://asuserwiki.atlassian.net/wiki/spaces/UO/pages/1543602177/Processing+DAC+data+with+CrysalisPro) and processed in *CrysalisPro*. Due to the change in κ and ϕ settings between runs 2 and 3, and runs 4 and 5, that required recentring, the data were integrated in three sets of two runs (1 and 2, 3 and 4, and 5 and 6), and then merged post-integration. Refinement of tHEWL was performed using *phenix.refine* (Afonine *et al.*, 2012 ▸) with Protein Data Bank (PDB) (Berman *et al.*, 2000 ▸) entry 2yvb as the starting model, and manual rebuilding using *Coot* (Emsley *et al.*, 2010 ▸). Refinement of anisotropic *B*-factors was restricted to seven translation–libration–screw groups (defined automatically by *phenix.refine*). Structure factors and final model coordinates are deposited at the PDB (ID 8f2g). The ambient structure of l-threonine was solved using the *ShelXT* (Sheldrick, 2015 ▸) structure solution program and using *Olex2* (Dolomanov *et al.*, 2009 ▸) as the graphical interface using the intrinsic phasing solution method. The model was refined using the 2018/3 version of *ShelXL* using full-matrix least-squares minimization on *F* ^2^. H-atom positions were calculated geometrically and refined using the riding model, except the hydroxyl and methyl H-atoms, where a difference electron density synthesis was calculated around the circle which represents the location of possible hydrogen positions. Once found, the hydroxyl and methyl H-atoms were constrained and refined until they converged (so-called AFIX 147 and 137 constraints). High-pressure laboratory and synchrotron data from the same sample of l-threonine were refined from the ambient-pressure coordinates using *Olex2 1.5*, and refined using the same procedure as above. Due to the reduced completeness of the datasets, thermal similarity restraints were applied to all non-H atoms.

## 4. Results and discussion

**Figure 3:**
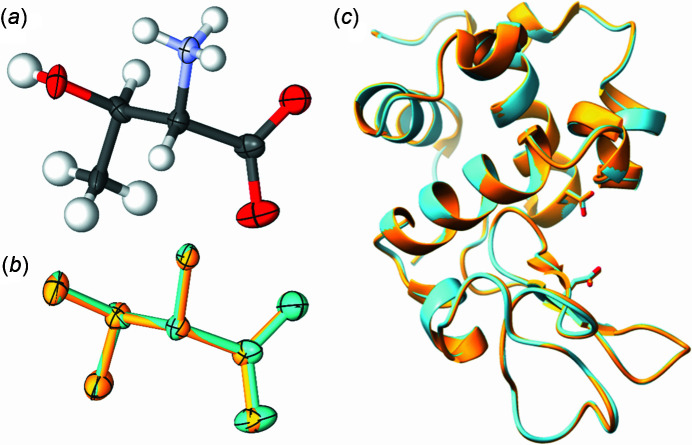
Connectivity and structure of l-threonine at 0.15 GPa collected using (*a*) the MX1 beamline at the Australian Synchrotron and (*b*) a superposition of the crystal structures of l-threonine at 0.15 GPa from a laboratory X-ray source (orange) and the MX1 beamline (light-blue) (H removed for clarity). A superposition of the structures of lysozyme determined at 0.44 GPa (current study – blue) and lysozyme determined at 0.1 MPa {PDB 4wld [Yamada *et al.* (2015 ▸) – orange]} are shown in (*c*). Colour scheme for (*a*) is: C – grey; O – red; N – blue; H – white. Ellipsoids are drawn at 50% probability.

Crystallographic data for l-threonine at ambient pressure, and at 0.15 GPa collected in-house on the laboratory diffractometer and at the synchrotron, and tHEWL at 0.44 GPa collected at the synchrotron have been summarized and shown in Table 2 ▸ and are given in Tables S1 to S4 of the supporting information. The crystallographic data for l-threonine collected in-house and on MX1 are unsurprisingly comparable; however, the data collected on MX1 is 678 times faster (14 s versus 9504 s). The unit-cell parameters for the same crystal of l-threonine at 0.15 GPa collected on a laboratory source and MX1 differ by less than 0.2%, and both similar data completeness and refinement statistics (Table 2 ▸), indicating the reliability of the high-pressure data collected on MX1. The data are also congruent with the same crystal of l-threonine characterized under ambient conditions, as well as with its previously published structure (Giordano *et al.*, 2019 ▸). Restraints were only required for thermal parameters, though we suspect this may not be the case for larger molecular systems. Nevertheless, the connectivity and refined structures were almost identical, as shown by a superposition of the structures in Fig. 3 ▸.

The crystal structure refinement of tHEWL at 0.44 GPa was also in good agreement with the previously published structures of tHEWL at 0.1 MPa (Yamada *et al.*, 2015 ▸) and at 0.1 GPa (Kundrot & Richards, 1987 ▸), with their unit-cell parameters differing by less than 0.6% despite the change in pressure. Comparable resolution was also achieved, with the mean *I*/σ(*I*) falling below 2.0 at a resolution of 2.10 Å, compared with 2.00 Å and 1.54 Å for the lower pressure structures. A superposition of tHEWL at 0.44 GPa and 0.1 MPa is shown in Fig. 3 ▸, showing almost identical structures.

Details on subsequent high-pressure protein data were published in 2002 by Fourme *et al.* (2002 ▸) though more recent studies have shown pressure to be a useful tool for exploring stability of milk proteins in order to develop new processing technologies in the dairy industry (Olsen *et al.*, 2022 ▸). Here, the high-pressure setup has been shown to be applicable to proteins, though further work will be needed in order to collect better data on protein crystals under pressure, and is an area ripe for exploration. Although limited by the energy of the synchrotron X-ray energies available, the setup on MX1 is ideal for high-throughput high-pressure single-crystal diffraction, and is arguably, the fastest static high-pressure single-crystal diffraction setup in the world.

## 5. Conclusions and perspectives

Here we have presented a new high-pressure single-crystal diffraction setup available for users at the Australian Synchrotron on the MX1 beamline for collecting molecular and protein crystal structures. A bespoke miniature diamond anvil cell (MMB cell) has been designed and implemented on the beamline which allows fast and reliable static pressure data collections with minimal reconfiguration of the beamline. Data collected on two samples, a small molecule (l-threonine) and a protein (tHEWL), have shown the efficacy of the beamline for collecting high-pressure single-crystal data. Impressively, equivalent data were collected on a single-crystal sample of l-threonine 679 times faster than using a laboratory source, while data collected on tHEWL shows promise for using the MX1 beamline for collecting high-pressure single-crystal diffraction data.

## 6. Related literature

The following references, not cited in the main body of the paper, have been cited in the supporting information: Karplus & Diederichs (2015 ▸); Pettersen *et al.* (2021 ▸).

## Supplementary Material

Crystal structure: contains datablock(s) ambient, threonine_150000kPa_lab, threonine_150000kPa. DOI: 10.1107/S160057752300406X/vy5009sup1.cif

Structure factors: contains datablock(s) ambient. DOI: 10.1107/S160057752300406X/vy5009ambientsup2.hkl

Structure factors: contains datablock(s) threonine_150000kPa_lab. DOI: 10.1107/S160057752300406X/vy5009threonine_150000kPa_labsup3.hkl

Structure factors: contains datablock(s) threonine_150000kPa. DOI: 10.1107/S160057752300406X/vy5009threonine_150000kPasup4.hkl

Supporting information file. DOI: 10.1107/S160057752300406X/vy5009threonine_150000kPasup5.cml

SI. DOI: 10.1107/S160057752300406X/vy5009sup6.pdf

CCDC references: 2227429, 2227430, 2227431

## Figures and Tables

**Table 1:** Summary of the high-pressure data collection strategy on MX1 for the MMB cell, with an opening half-angle of 40° All runs were collected as ω-scans. 2θ is fixed at 0° due to the large area of the Dectris EIGER 2 9M detector. The detector distance is 105 mm from sample to detector.

Run	Range of ω (°)	κ (°)	ϕ (°)
1	−35 to 35	0	0
2	145 to 215	0	0
3	−35 to 35	180	0
4	145 to 215	180	0
5	−35 to 35	180	180
6	145 to 215	180	180

**Table 2:** Selective refinement statistics for L-threonine and tHEWL collected using laboratory and synchrotron data Comparable laboratory and synchrotron data are only available for L-threonine.

	L-Threonine				tHEWL		
X-ray source	Synergy-S	Synergy-S	MX1	LTHREO21†	MX1	3LYM‡	4WLD§
Pressure (GPa)	Ambient	0.15	0.15	Ambient	0.44	0.1	0.0001
Completeness (%)	100	51.2	44.7	100	63.2	N/A	N/A
Resolution	0.73	0.80	0.76	N/A	2.10	2.00	1.54
*I*/σ	26.7	31.9	54.6	N/A	7.0	N/A	N/A
*R*_1_ (%)	3.95	4.04	4.04	2.60	21.7	14.9	15.0
Data collection time (s)	2337	9504	14	N/A	70	N/A	N/A
Space group	*P*2_1_2_1_2_1_	*P*2_1_2_1_2_1_	*P*2_1_2_1_2_1_	*P*2_1_2_1_2_1_	*P*4_3_2_1_2	*P*4_3_2_1_2	*P*4_3_2_1_2
*a* (Å)	5.1456 (2)	5.1419 (2)	5.1498 (5)	5.1481 (1)	79.045	78.69	79.197
*b* (Å)	7.7415 (4)	7.7217 (4)	7.7304 (5)	7.7426 (1)	79.045	78.69	79.197
*c* (Å)	13.6223 (6)	13.613 (5)	13.602 (6)	13.6138 (2)	37.865	38	37.9
*V* (Å^3^)	542.64 (4)	540.5 (2)	541.4 (3)	542.642 (15)	236585	235300	237715

† CSD reference = LTHREO21 (Giordano *et al.*, 2019 ▸).

‡ PDB reference = 3lym (Kundrot & Richards, 1987 ▸).

§ PDB reference = 4wld (Yamada *et al.*, 2015 ▸).
