# Supplementary material for: High-pressure single-crystal diffraction at the Australian Synchrotron
Source: J Synchrotron Radiat. 2023 Jun 15;30(Pt 4):841–6. doi: 10.1107/S160057752300406X (PMC10325021; doi:10.1107/S160057752300406X)
Supplement: Supplementary file 6 [file s-30-00841-sup6.pdf]

JOURNAL OF SYNCHROTRON RADIATION

Volume 30 (2023)

Supporting information for article:

High-Pressure Single-Crystal Diffraction at the Australian Synchrotron

Stephanie A. Boer, Jason R. Price, Alan Riboldi-Tunnicliffe, Rachel Williamson, Aston Summers, Gemma F. Turner, Isabelle Jones, Charles Bond, Alice Vrielink, Andrew C. Marshall, John Hitchings and Stephen A. Moggach

S1. Crystallization conditions, data processing and refinement of Lyophilised chicken egg white Lysozyme (THEWL)

THEWL (Sigma-Aldrich) was dissolved to 40 mg/mL in 20mM sodium acetate pH 4.7. Tetragonal crystals were grown by vapour diffusion, as described previously [REF: 10.1107/s1399004715000292] (**Table S1**). Diffraction data were processed in space group $P4_32_12$ (**Table S2**). Refinement of atomic coordinates was performed using *phenix.refine (Afonine et al., 2012)* with PDB 2yvb as the starting model, and manual rebuilding using Coot (Emsley *et al.*, 2010) (**Table S3**). Refinement of anisotropic *B*-factors was restricted to seven translation-libration-screw groups (defined automatically by *phenix.refine*). Structure factors and final model coordinates are deposited at the Protein Data Bank (dataset ID: D_1000268609 and PDB ID: 8F2G). To calculate RMSD across all α-carbon pairs, superposition of the current structure with 4wld was performed using UCSF ChimeraX (v1.3) (Pettersen *et al.*, 2021) *Matchmaker* tool, with iterative pruning of long atom pairs switch off. Superposition with the crystal structure of lysozyme previously determined at atmospheric pressure (0.1 MPa) (Yamada *et al.*, 2015) reveals no significant structural changes; RMSD between 129 atom pairs is 0.154 Å.

Table S1 Crystallization conditions

Method	Vapour diffusion, hanging drop
Plate type	Hampton Research VDX™ Plate (24-well) with siliconized glass cover-slips
Temperature (K)	293
Protein concentration	40 mg/mL
Buffer composition of protein solution	20 mM Na acetate pH 4.7
Composition of reservoir solution	1 M NaCl, 100 mM Na acetate pH 4.7
Volume and ratio of drop	6 µL at 1:1 ratio of reservoir:protein solution
Volume of reservoir	1 mL

Table S2 Data collection and processing. Values for the outer shell are given in parentheses. Data were collected from a single crystal.

Wavelength (Å)	0.71092
Space group	$P4_32_12$
a, *c* (Å)	79.045, 37.865
α, β, γ (°)	90, 90, 90
Mosaicity (°)	0.06
Resolution range (Å)	55.9–1.84 (1.88–1.84)
Total No. of reflections	31390 (1609)
No. of unique reflections	6894 (383)
Completeness (%)	63.2 (57.3)
Redundancy	4.6 (4.2)
$\langle I/\sigma(I) \rangle$	7.0 (0.7) #
R_{meas}	0.124 (1.557)
$R_{p.i.m.}$	0.055 (0.708)
$CC_{1/2}$	0.994 (0.440)
Overall *B* factor from Wilson plot (Å^2)	19.6

Mean $I/\sigma(I)$ falls below 2.0 at a resolution of 2.10 Å. High resolution cut-off for data processing was selected primarily by reference to $CC_{1/2}$ as recommended by (Karplus & Diederichs, 2015).

Table S3 Structure refinement of THEWL. Values for the outer shell are given in parentheses.

Resolution range (Å)	35.35 - 1.842 (1.908 - 1.842)
Completeness (%)	63.10 (57.74)
No. of reflections, working set	6547 (593)
No. of reflections, test set	303 (26)
Final R_{work}	0.217 (0.334)
Final R_{free}	0.263 (0.337)
No. of non-H atoms	
Protein	993
Water	44
Total	1037
R.m.s. deviations	
Bonds (Å)	0.0023
Angles (°)	0.57
Average *B* factors (Å^2)	
Protein	28.04
Water	29.18
Ramachandran plot	
Most favoured (%)	96.85
Allowed (%)	3.15

Table S4 L-threonine Crystallographic Experimental details. For all structures: $C_4H_9NO_3$, M_r = 119.12, orthorhombic, $P2_12_12_1$, Z = 4. Experiments were carried out at 293 K. H-atom parameters were constrained. CCDC 2227429-2227431 contain the supplementary crystallographic data. The data can be obtained free of charge from The Cambridge Crystallographic Data Centre via http://www.ccdc.cam.ac.uk/data_request/cif.

Data set	L-threonine under ambient conditions	L-threonine at 0.15 GPa (laboratory data)	L-threonine at 0.15 GPa (synchrotron data)
Crystal data			
a, *b*, *c* (Å)	5.1456 (2), 7.7415 (4), 13.6223 (6)	5.1419 (2), 7.7217 (4), 13.613 (5)	5.1489 (5), 7.7304 (5), 13.602 (6)
V (Å^3)	542.64 (4)	540.49 (19)	541.4 (3)
Radiation type	Mo *K*a	Mo *K*a	Synchrotron, l = 0.71073 Å
m (mm^{-1})	0.12	0.13	0.13
Crystal size (mm)	0.2 × 0.1 × 0.1	0.2 × 0.2 × 0.1	0.2 × 0.2 × 0.1
Data collection			
Diffractometer	XtaLAB Synergy, Single source at home/near, HyPix	XtaLAB Synergy, Single source at home/near, HyPix	MX1
Absorption correction	Multi-scan *CrysAlis PRO* 1.171.41.115a (Rigaku Oxford Diffraction, 2021) Empirical absorption correction using spherical harmonics, implemented in SCALE3 ABSPACK scaling algorithm.	Multi-scan *CrysAlis PRO* 1.171.41.123a (Rigaku Oxford Diffraction, 2022) Empirical absorption correction using spherical harmonics, implemented in SCALE3 ABSPACK scaling algorithm.	Multi-scan *SADABS2012*/1 (Bruker,2012) was used for absorption correction.
T_{min}, T_{max}	0.706, 1.000	0.203, 1.000	0.579, 0.745
No. of measured, independent and observed [I > 2s(I)] reflections	5981, 1304, 1110	7737, 567, 482	1209, 468, 428
R_{int}	0.044	0.059	0.013
(sin q/l)$_{max}$ (Å^{-1})	0.682	0.625	0.654
Refinement			
$R[F^2 > 2s(F^2)]$, $wR(F^2)$, S	0.040, 0.104, 1.08	0.040, 0.125, 1.13	0.040, 0.142, 1.22

No. of reflections	1304	567	468
No. of parameters	76	76	70
No. of restraints	0	48	48
$D\rho_{max}$, $D\rho_{min}$ (e Å^{-3})	0.25, -0.21	0.17, -0.17	0.22, -0.22
Absolute structure	Flack x determined using 383 quotients [(I+)-(I-)]/[(I+)+(I-)] (Parsons, Flack and Wagner, Acta Cryst. B69 (2013) 249-259).	Flack x determined using 170 quotients [(I+)-(I-)]/[(I+)+(I-)] (Parsons, Flack and Wagner, Acta Cryst. B69 (2013) 249-259).	Flack x determined using 157 quotients [(I+)-(I-)]/[(I+)+(I-)] (Parsons, Flack and Wagner, Acta Cryst. B69 (2013) 249-259).
Absolute structure parameter	-0.2 (10)	-0.6 (10)	-0.2 (10)

References

Afonine, P. V., Grosse-Kunstleve, R. W., Echols, N., Headd, J. J., Moriarty, N. W., Mustyakimov, M., Terwilliger, T. C., Urzhumtsev, A., Zwart, P. H. & Adams, P. D. (2012). *Acta Cryst.* **D68**, 352-367.

Emsley, P., Lohkamp, B., Scott, W. G. & Cowtan, K. (2010). *Acta Cryst.* **D66**, 486-501.

Karplus, P. A. & Diederichs, K. (2015). *Curr. Opin. Struct. Biol.* **34**, 60-68.

Pettersen, E. F., Goddard, T. D., Huang, C. C., Meng, E. C., Couch, G. S., Croll, T. I., Morris, J. H. & Ferrin, T. E. (2021). *Protein Sci.* **30**, 70-82.

Yamada, H., Nagae, T. & Watanabe, N. (2015). *Acta Cryst.* **D71**, 742-753.
